# Stan and BART for Causal Inference: Estimating Heterogeneous Treatment Effects Using the Power of Stan and the Flexibility of Machine Learning

**DOI:** 10.3390/e24121782

**Published:** 2022-12-06

**Authors:** Vincent Dorie, George Perrett, Jennifer L. Hill, Benjamin Goodrich

**Affiliations:** 1 Code for America, San Francisco, CA 94103, USA; 2 Department of Applied Statistics, Social Science, and the Humanities, New York University, New York, NY 10003, USA; 3 Department of Political Science, Columbia University, New York, NY 10025, USA

**Keywords:** BART, Stan, causal inference, machine learning, heterogeneous treatment effects, multilevel data, grouped data

## Abstract

A wide range of machine-learning-based approaches have been developed in the past decade, increasing our ability to accurately model nonlinear and nonadditive response surfaces. This has improved performance for inferential tasks such as estimating average treatment effects in situations where standard parametric models may not fit the data well. These methods have also shown promise for the related task of identifying heterogeneous treatment effects. However, the estimation of both overall and heterogeneous treatment effects can be hampered when data are structured within groups if we fail to correctly model the dependence between observations. Most machine learning methods do not readily accommodate such structure. This paper introduces a new algorithm, stan4bart, that combines the flexibility of Bayesian Additive Regression Trees (BART) for fitting nonlinear response surfaces with the computational and statistical efficiencies of using Stan for the parametric components of the model. We demonstrate how stan4bart can be used to estimate average, subgroup, and individual-level treatment effects with stronger performance than other flexible approaches that ignore the multilevel structure of the data as well as multilevel approaches that have strict parametric forms.

## 1. Introduction

Causal effects represent comparisons between outcomes in factual and counterfactual worlds. That is, for each observation in a study, we need to be able to not only measure the outcome the subjects experienced under the treatment regime they were exposed to, we also have to predict what their outcome *would have been* in a counterfactual world where they were exposed to a different treatment. Since we have no data from the counterfactual world, estimation in causal inference requires solving a difficult missing data problem. In the absence of a randomized experiment, this is often approached by conditioning treatment effect estimates on many pretreatment covariates in an attempt to ensure that estimates have adjusted for any relevant differences across groups. An increasing number of causal inference strategies approach this missing data problem (implicitly or explicitly) by predicting these missing outcomes using flexible machine learning algorithms (for example, [1,2,3,4,5,6,7]).

In many causal inference settings, we additionally expect that data will have a grouped structure. For instance, we might have measurements of students within schools, patients within hospitals, or individuals incarcerated within institutions. In such settings, observations may have correlated error structures within these groups. We may also have reason to believe that the impact of the treatment exposure will vary across these groups. Most current machine-learning-based causal inference strategies either ignore such error structure or assume that errors are independently and identically distributed. Moreover, while these approaches may allow for estimation of treatment effect heterogeneity across groups they typically do so inefficiently and fail to capitalize on a potential distribution for these varying effects. In essence, typically these algorithms can at best accommodate a fixed effects approach to groups rather than a random effects approach.

On the other hand, multilevel models, in various forms, have been used for decades to accommodate grouped error structures and to efficiently estimate varying treatment effects [8,9,10]. This approach has been particularly successful in the context of randomized experiments where the concern about appropriately incorporating covariates is minimized for two key reasons. The most primal reason is that in a completely randomized experiments, we do not need to condition on covariates at all to obtain unbiased estimates of treatment effects. However, even if we do fit a model conditional on covariates to experimental data (for example, to achieve greater efficiency), treatment effects estimates should be relatively robust to model misspecification due to the fact that common support across treatment groups is ensured in expectation [9,11].

This paper introduces a multilevel machine-learning-based approach to causal effect estimation that combines the strengths of these two existing modeling frameworks. It builds on an established machine learning algorithm, Bayesian Additive Regression Trees (BART; [12,13]), that provides a flexible fit to the relationship between the outcome and the covariates. The traditional form of the model is extended, however, to include a parametric component that allows for covariates to be included with explicit parametric forms and additionally allows group-level deviations from the common parameters to be modeled with a hierarchical structure. The Markov chain Monte Carlo algorithm in Stan is used to draw the unknowns in the parametric component and the hierarchical structure, given the trees, and the BART algorithm is used to draw the trees, given the parametric and hierarchical components. This Gibbs sampling algorithm is, to our knowledge, the first to combine BART and Stan updates.

## 2. Background and Context

This section provides some background and a summary of the building blocks of our new algorithm, stan4bart. It also discusses other approaches to causal inference for heterogeneous treatment effect estimation in settings with grouped data.

### 2.1. BART

Bayesian Additive Regression Trees (BART; [12,13]) is a Bayesian machine learning algorithm that can provide a flexible fit for a wide variety of conditional expectations of the general form E[Y∣X], where *Y* denotes the outcome of interest and *X* represents a vector of covariates or predictors. The standard BART algorithm has been implemented in several packages including BayesTree, bartMachine, and BART. We focus on the dbarts [14] software package because it has an efficient implementation of the base BART algorithm and was explicitly designed to incorporate model extensions of the kind described in this paper.

While the standard BART implementation assumes a continuous response and normal, independent, and identically distributed errors, many extensions have been proposed. One of the original BART papers describes a variation for a binary response based on a probit link [13]. Extensions of this implementation capitalize on better priors or the use of cross-validation to choose hyperparameters for the default priors resulting in better performance (for example see [15]). Subsequent work has extended BART for a wide variety of different regression models for categorical, count, zero-inflated, multivariate, and right-censored survival responses [16,17,18,19,20].

### 2.2. BART for Causal Inference

BART has been proposed as a strategy for estimating causal effects [1]. The basic idea is to use the algorithm to fit E[Y∣X,Z] in a way that minimizes assumptions about the parametric relationships between the outcome, *Y*, and the covariates, *X*, while allowing that relationship to vary across treatment groups defined by *Z*. This provides a flexible approach to making predictions about missing counterfactual values (for example, the outcome a participant would be predicted to experience under a different treatment regime) based on the observed covariates. Moreover, this approach allows for the estimation of posterior predictive distributions for each potential outcome, which enables the formation of coherent uncertainty intervals both for potential outcomes and causal effects. The use of BART for causal inference is explained in more detail later in the paper.

BART has been shown to have strong performance relative to standard parametric models as well as a variety of machine learning approaches to regression [1,12,15,21,22,23,24,25]. Functions to facilitate the use of BART for causal inference have been implemented in the bartCause function (with dbarts at its foundation), which is described in more detail in Section 5.1.

### 2.3. Causal Inference with Multilevel Data

The standard BART model assumes that error terms are independently, identically, and normally distributed, which limits its applicability. Extensions have been proposed to accommodate heteroskedasticity in error terms and non-Gaussian response variables [17,20,26]. However, none of these approaches allow for a dependence between error terms. Bisbee [27] used BART in an explicitly multilevel setting, however, groups were only incorporated as fixed effects, and thus no direct correlations were modeled. Zeld et al. [6] fit a semiparametric model with an arbitrary linear term, but no multilevel component. Moreover, Hahn et al. [7] proposed an extension of BART for causal inference, Bayesian causal forests (BCF), which has advantages for estimating heterogeneous treatment effects. In the standard implementation, however, the errors were assumed to be independent. Multilevel extensions to BCF (random intercepts and varying slopes on treatment assignment) have been used in applied work [28,29,30] but no software has been made available.

Suk and Kang [31] fit models that are in some ways conceptually similar to those in stan4bart, with arbitrarily complex, machine learning components as well as parametric, linear ones. However, their primary aim was to produce consistent estimates in the presence of unmeasured, group-level confounders and as such, their approach addressed a different issue. Another related BART extension was described in Spanbauer and Sparapani [32]. This approach incorporated random effects for longitudinal repeated measures into the BART model as well as subject clustering within groups.

As a precursor to stan4bart, BART with varying intercepts was implemented as rbart_vi in the dbarts package. It was also independently developed in [33]. stan4bart allows for more general multilevel structures. This paper compares the performance of traditional BART with rbart_vi and stan4bart, as well as several other options.

## 3. Notation, Estimands, and Assumptions

We formalize our model and assumptions relying on the Rubin–Neyman causal model [34,35]. For simplicity, we focus on situations with a binary treatment variable, *Z*. Exposure to $Z_{i}$ for observation *i* allows the potential outcome under treatment, $Y_{i}\left(Z_{i}=1\right)\equiv Y_{i}\left(1\right)$, to manifest. A lack of exposure (or possibly exposure to a different treatment modality) leads to the expression of the other potential outcome $Y_{i}\left(Z_{i}=0\right)\equiv Y_{i}\left(0\right)$. The observed outcome $Y_{i}=Y_{i}\left(0\right)\times \left(1-Z_{i}\right)+Y_{i}\left(1\right)\times Z_{i}$ is thus a function of the potential outcomes and the treatment assignment. Even though we focus on group-structured data, this article only considers situations where treatment assignment occurs at the individual level.

### 3.1. Estimands

In our framework, several estimands are of interest. In this section, we index observations by *i* and refrain from further indexing by groups as this is unnecessary for our purposes and merely clutters the notation. We start by defining an individual-level causal effect on unit *i* as $\tau_{i}=Y_{i}\left(1\right)-Y_{i}\left(0\right)$. The estimand is rarely an inferential goal because it is not identifiable without extremely strong assumptions [36]. However, the individual-level causal effect is a building block for many common causal estimands, which can be expressed as averages of this estimand over different subsamples.

Consider, for instance, the sample average treatment effect (SATE) which takes an average of these individual effects over the entire sample, $\mathrm{SATE}=\frac{1}{N}\sum_{i}^{N}\tau_{i}$, where *N* denotes the size of our analytic sample. In observational studies we often care more about estimating the average treatment effect for those who we observe to self-select into a treatment or program, or conversely on those who have not yet had access to a treatment or program. These concepts map more closely to estimands referred to as the effect of the treatment on the treated or the effect of the treatment on the controls. This paper focuses on the former quantity measured for our sample. This estimand, the sample average treatment effect on the treated (SATT), can be formalized as $\mathrm{SATT}=\frac{1}{N_{t}}\sum_{i}^{N}\tau_{i}I\left(Z_{i}=1\right)$, where $N_{t}=\sum_{i}^{N}Z_{i}$ is the number of people in the treatment group. It is worth noting, however, that BART and stan4bart can be used to estimate population and conditional versions of these estimates as well [1].

Researchers with access to observational multilevel data might also be curious to explore whether treatment effects vary over the groups that define the multilevel data structure. Thus, we also explore the performance of our estimation strategy with regard to group-level causal estimands that can capture the heterogeneity in average treatment effects across groups (such as hospitals, schools, or counties). If we use g[i] to denote the group membership of person *i*, we can define a group-level sample average treatment effect for group *g* as $\mathrm{GSATE}(g)=\frac{1}{n_{g}}\sum_{i}^{N}\tau_{i}I\left(g\left[i\right]=g\right)$, where $n_{g}=\left|\left\{i:g\left[i\right]=g\right\}\right|$ denotes the sample size in group *g*. A group-level analog to the SATT is thus the group-level sample average treatment for group *g* among the treated, $\mathrm{GSATT}(g)=\frac{1}{n_{g}^{1}}\sum_{i}^{N}\tau_{i}I\left(g\left[i\right]=g\right)I\left(Z_{i}=1\right)$. Here, $n_{g}^{1}$ denotes the number of treated observations in group *g* such that $n_{g}^{1}=\left|\left\{i:g\left[i\right]=g,Z_{i}=1\right\}\right|$.

To understand the treatment effect heterogeneity at a more fine-grained level it would help to be able to estimate individual-level causal effects directly. Since $\tau_{i}$ is generally not identifiable without extreme assumptions, researchers increasingly focus instead on the conditional average treatment effect function, $\mathrm{CATE}(x)=E[\tau_{i}|X_{i}=x_{i}]$. An important property of the CATE is that the estimator with the smallest mean squared error (MSE) for CATE will also have the smallest MSE for the individual causal effect, $\tau_{i}$ [4]. If we can obtain accurate estimates of the CATE across the instantiations of the covariate values defined in our sample, it will allow us to explore the treatment effect heterogeneity more flexibly (see, for instance, [37]). Henceforth, we refer to each CATE that reflects the covariate values specific to an individual in our sample as an iCATE; the collection of these for our sample is referred to as the iCATEs for our sample.

### 3.2. Assumptions

The BART and stan4bart approaches to causal inference yield unbiased estimates only if several assumptions are satisfied. The first assumption requires that we have measured all confounders for the effect of *Z* on *Y*. This so-called unconfoundedness, or ignorability, assumption can be formalized as Y(0),Y(1)⊥Z∣X [34], where *X* denotes all measured pretreatment covariates in our analysis, both at the individual and group level (we drop the subscripts here for convenience). The intuition behind this assumption is that it allows us to use information from observations in one treatment condition to help make predictions about the other counterfactual outcome of a similar observation in a different treatment condition. Here, similarity is defined by the covariates. This is generally considered to be a strong assumption and it is untestable. For strategies to address potential violations of this assumption see, for instance, Dorie et al. [23], Carnegie et al. [38].

If for a given individual no similar observations exist that received a different treatment, it may be challenging to make a prediction for that individual’s potential outcome under that different treatment. Therefore, we additionally make an assumption that all neighborhoods of the covariate space with observations have a nonzero probability of having both treated and control observations. This is often referred to as an overlap or common support assumption and can be formalized as 0<Pr(Z=z∣X)<1. If this assumption fails to hold, a general strategy is to identify which observations lack empirical counterfactuals. Several BART-based strategies have been developed to identify and discard these observations [22] and there is evidence that these perform better than traditional propensity score strategies.

Our definition of potential outcomes above implicitly assumed that the only treatment assignment that is necessary to define the potential outcomes for observation *k* is the treatment received by that observation, $Z_{k}$. Moreover, for a treatment effect estimand to have meaning, we must assume that the treatment assigned to each of the different observations and referred to as *Z* takes only one form. As a crude example, it would not make sense to define an estimand with weight loss intervention, *Z*, if $Z_{a}$ refers to a drug and $Z_{b}$ refers to an exercise regime. These assumptions are often jointly referred to as the stable unit treatment value assumption (SUTVA) [39]. While studies can be designed to increase the plausibility of SUTVA, researchers often have access to data where they do not have this type of control over the study design and rather hope that it holds approximately. To decrease the complexity of the issues addressed in this paper, we assume that SUTVA holds.

When these structural assumptions hold, then E[Y(0)∣X]=E[Y∣Z=0,X] and E[Y(1)∣X]=E[Y∣Z=1,X]. That means that our task as data analysts can be reduced to a modeling task. Our goal then is to reduce the parametric assumptions required to estimate these conditional expectations and appropriately reflect our uncertainty about these estimates. The proposed algorithm is intended to provide robust inference in this setting.

## 4. Combining Stan and BART: stan4bart

This section describes how Stan [40] and BART are integrated to form a new modeling strategy. Since this section focuses on modeling strategies for observed data, we now use lower-case letters for observed covariates, *x*, and treatment assignment, *z*, when we condition on these in our model. When it is desired to extrapolate the following results to population level quantities, *X* can once more be treated as a random variable.

### 4.1. Stan and Variations on the No-U-Turn Sampler

One of the original motivations [41] for developing the MCMC algorithm in Stan was to draw from the posterior distribution of multilevel models more efficiently than the pure Metropolis–Hastings and Gibbs sampling algorithms that preceded it. Pure Metropolis–Hastings algorithms often have an optimal acceptance probability below 0.25, implying that only about one in four MCMC iterations move from the previous state and that the mixing is slow. Gibbs samplers draw a unique value of each parameter (block) from its full-conditional distributions, but when the variance of the full-conditional distribution is small, they do not move very far from the previous state.

Stan has not relied on the algorithm described in [41] since the release of version 2.10 in 2016, but its current performance is at least as good [42]. Hamiltonian MCMC algorithms, like the one in Stan, work by analogy to Hamiltonian physics [41,43]. The vector of unknown *location* parameters is augmented with a vector of *momentum* parameters of the same size. These momentum parameters are assumed to be independent, and each has a Gaussian prior with mean zero and a standard deviation that is tuned during the warm-up phase of the algorithm. Since the momentum parameters do not enter the likelihood function, their posterior distribution is the same as their prior distribution. However, the realizations of the momentum parameters serve as a catalyst to provide an initial push to the location parameters that moves them through a parameter space whose topology is defined by the log-likelihood function with the logarithm of the probability density functions (PDFs) specified for the prior on the location parameters. The location parameters continue to evolve forward (that is, with the momentum realization) and backward (that is, opposite the momentum realization) in time until the Euclidean distance between the forward-moving and backward-moving location parameters starts to shrink, at which point a *U-turn* is declared and a realization of the location parameters is taken from the footprints they made along their journey via multinomial sampling with products of Metropolis-like acceptance probabilities. However, unlike pure Metropolis–Hasting algorithms, the algorithm in Stan yields an acceptance probability that is usually very close to 1. The realized parameter vector is then used as the starting point for the next iteration when a new realization of the momentum parameters is obtained.

As a result—and unlike both Gibbs sampling and pure Metropolis–Hastings algorithms— the first-order autocorrelation between consecutive realizations of a parameter tends to be negative with Stan and the autocorrelations at higher lags tend to dissipate quickly. The formula for effective sample size used by Stan is $\frac{S}{1+\sum_{j=1}^{\infty }\rho_{j}}$, where *S* is the nominal number of MCMC draws and $\rho_{j}$ is the *j*-th order autocorrelation between draws that are separated by *j* steps. If the first-order autocorrelation is sufficiently negative, then the denominator is less than 1, and the estimator of the mean is better than would be obtained from independent draws even if it were possible to obtain independent draws.

### 4.2. Stan for Multilevel Models

Our work seeks to augment the BART model with a grouped error structure such as those found in more traditional multilevel models. We review that framework first.

A general, linear, multilevel model for one observation can be written as

(1) $$\begin{array}{cc} Y|\vec{\beta },\vec{\lambda },\epsilon & =x^{\beta }\vec{\beta }+w\vec{\lambda }+\epsilon ,\\ \vec{\lambda }|\Sigma_{\lambda } & ∼N(0,\Sigma_{\lambda }),\\ \epsilon |\sigma & ∼N(0,\sigma^{2}). \end{array}$$

Here, $\vec{\beta }$ is a traditional linear, parametric vector of coefficients and $x^{\beta }$ is a standard linear model design vector. The first element of $x^{\beta }$ is often the constant “1”, so that the first element of $\vec{\beta }$ enters the model as an offset or baseline. $\vec{\lambda }$ is the vector of all parametric random intercepts and slopes and *w* is a sparse vector which serves to select out and weight the appropriate random values, essentially containing group-level dummy variables and interactions between the group-level dummy variables and other predictors in $x^{\beta }$. To imply the correct covariance structure, $\Sigma_{\lambda }$ consists of block-diagonal repetitions of the covariance matrices of the values for one or more grouping factors. For an explanation of the design of $\vec{\lambda }$, *w*, and $\Sigma_{\lambda }$, see [44]. Finally, the errors $\left(\epsilon \right)$ are independent of the group variation and normally distributed with an expectation of zero and a variance of $\sigma^{2}$. All inference is conditional on both the $x^{\beta }$ and *w* vectors.

The Bayesian version of such a model fit by Stan—and extended by stan4bart— includes prior distributions for $\vec{\beta }$, $\Sigma_{\lambda }$, and σ (or $\sigma^{2}$). The prior distribution on the covariance matrix, $\Sigma_{\lambda }$, can be rather consequential but rarely do researchers have strong beliefs about it. It is now commonplace when using Stan to decompose covariance matrix as Σ=DCD, where *D* is a diagonal matrix of standard deviations and *C* is a correlation matrix. The prior on the standard deviations is fairly easy to specify, as any proper distribution for positive random variables would do and even improper ones often work fine. By default, the prior on the standard deviations is an exponential prior, which has maximum entropy among positive random variables with a given expectation. The prior on the correlation matrix—if it has more than one row and column—is jointly uniform by default over all symmetric, positive definite matrices that have ones along their diagonal. This LKJ prior for correlation matrices is used the vast majority of the time in Stan programs, but a shape hyperparameter can be specified to a value greater than 1 to concentrate on the identity matrix [45]. A shape hyperparameter value between zero and one is mathematically possible, which would make sense if the identity matrix were thought to be the least likely correlation matrix rather than the prior mode.

Unlike frequentist estimators of multilevel models [44] that integrate $\vec{\lambda }$ out of the original likelihood function to form a new likelihood function that can be maximized with respect to $\vec{\beta }$ and the group-level (co-)variances only, the Bayesian approach can—and in our case, does—condition on the group-level structure defined by *w* and draws posterior realizations of $\vec{\lambda }$ jointly along with the other parameters. In our experience, maximizing the integrated likelihood function often yields an optimum on the boundary of the parameter space where some diagonal element of the error covariance matrix is zero or the covariance matrix is otherwise numerically singular. Bates et al. [46] report a similar experience with such models in the field of psychological linguistics but recommends eliminating variance components until numerical maximization is reliable and substantively useful. This problem is avoided automatically with MCMC and proper priors that constrain all the draws to be on the interior of the parameter space to yield good estimates of posterior means, medians, and quantiles, even if the posterior mode might be on the boundary of the parameter space.

### 4.3. Bayesian Additive Regression Trees

The BART algorithm consists of two pieces: a sum-of-trees model and a regularization prior. We describe the algorithm in a slightly extended way as compared to the original paper [12] to distinguish between the treatment variable, *z*, and the rest of the predictors, *x*. For a response variable *Y* ranging continuously between −0.5 and 0.5, a treatment variable *z*, and predictors *x*, we describe the sum-of-trees model by $Y=f(x,z;\vec{T},\vec{M})+\epsilon$, where $\epsilon ∼N(0,\sigma^{2})$ and $f\left(x,z;\vec{T},\vec{M}\right)=g\left(x,z;T_{1},M_{1}\right)+g\left(x,z;T_{2},M_{2}\right)+\cdots +g\left(x,z;T_{m},M_{m}\right)$. $\left(T_{j},M_{j}\right)$ defines a single regression tree submodel where $T_{j}$ is the tree topology and branching rules, $M_{j}$ are constants associated with each leaf node, and $g(x,z;T_{j},M_{j})$ is a function that uses $T_{j}$ to map (x,z) to a value in $M_{j}$. The number of trees is typically allowed to be large (Chipman et al. [12,13] originally suggested 200, though some recent work suggests that 50 may be sufficient [47], and in practice this number should not exceed the number of observations in the sample). As is the case with related sum-of-trees strategies (such as boosting), the algorithm requires a strategy to avoid overfitting. With BART this is achieved through a regularization prior that allows each $\left(T_{j},M_{j}\right)$ tree to contribute only a small part to the overall fit. BART fits the sum-of-trees model using an MCMC algorithm that cycles between draws of $\left(T_{j},M_{j}\right)$ conditional on σ and draws of σ conditional on all of the $\left(T_{j},M_{j}\right)$. Convergence can be monitored by plotting the residual standard deviation σ over time, though in general it makes sense to choose a statistic more relevant to one’s inferential goals.

The BART prior works to avoid overfitting by specifying distributions that help control the size of each tree, the shrinkage applied to the fit from each tree, and the uncertainty associated with the residual standard error. Interested readers can find more information on the model, prior, and fitting algorithms in Chipman et al. [12,13]. The key point is that BART can be used to flexibly fit even highly nonlinear response surfaces, which is consistent with our goal to fit E[Y(1)∣x]−E[Y(0)∣x] without making undue parametric assumptions.

Finally, we note that binary outcomes can be modeled by fixing σ to 1 and treating *Y* as a latent variable where $Y^{\prime }=I\left\{Y>0\right\}$ is observed.

### 4.4. stan4bart

For an arbitrary continuous response variable, stan4bart augments the multilevel model above by fitting the following, conditioned on covariates:

(2) $$\begin{array}{cc} Y|\vec{\beta },\vec{\lambda },\epsilon ,\vec{M},\vec{T} & =x^{\beta }\vec{\beta }+f\left(x,z;\vec{M},\vec{T}\right)+w\vec{\lambda }+\epsilon ,\\ \vec{\lambda }|\Sigma_{\lambda } & ∼N(0,\Sigma_{\lambda }),\\ \epsilon |\sigma & ∼N(0,\sigma^{2}). \end{array}$$

This model differs from that of Equation (1) in the inclusion of $f(x;\vec{M},\vec{T})$, a nonparametric sum-of-trees fit by BART (note that this component may or may not include *z*, we keep the term in the model for generality). The same latent variable formulation that allows BART to fit binary outcomes applies here.

The two sets of covariates, *x* and $x^{\beta }$, are integrated into a single model by first eliminating the global intercept term from $x^{\beta }$. Instead, for continuous outcomes the prior over the mean is explicitly set to the midpoint of the range of the response, and for binary outcomes it is set to 0.5. Shrinkage to different values in the binary case is supported by manually supplying a constant on the probit scale, as in [13]. In addition, having two design vectors raises the practical question when specifying a model of choosing which variables are included in each set. We discuss this at greater length below in Section 4.5; however, at this point it is sufficient to say that *x* and $x^{\beta }$ can share components without restriction.

At a high level, the model is implemented as a Gibbs sampler [48]. The parametric components given the nonparametric one are jointly sampled using a Hamiltonian Monte Carlo, no-U-turn sampler with a diagonal Euclidean adaptation matrix [41,42] and the converse is sampled sequentially through trees using the original BART’s *Bayesian backfitting* approach [12]. As discussed above, $\vec{\beta }$, σ, and $\Sigma_{\lambda }$ are all given priors and are included in the parametric sampling step.

In practical terms, this is accomplished by modifying and compiling into C++ a parametric Stan model that fits the above equation, with $f(x,z;\vec{M},\vec{T})$ treated as a generic linear *offset*, that is, a fixed value that shifts the mean of the response. The model itself is adapted from those used in the rstanarm package, a collection of model fitting functions implemented in Stan for the R programming language [49]. This C++ code is encapsulated in a custom mutable Stan sampler object which is coupled with a BART sampler set to have a fixed variance parameter and an offset term of its own. Using a “${\mathrm{vec}}_{i}\cdot$” operator to denote a vector that comprises i=1,…,N scalar values to run the Stan sampler collects the current draws of the BART sum-of-trees predictions for all observations into ${\mathrm{vec}}_{i}f\left(x_{i},z_{i};\vec{M},\vec{T}\right)$. It uses these to produce a draw of $\vec{\beta },\vec{\lambda },\sigma ,\Sigma_{\lambda }|\vec{Y},{\mathrm{vec}}_{i}f\left(x_{i},z_{i};\vec{M},\vec{T}\right)$. From this, σ and ${\mathrm{vec}}_{i}\left[x_{i}^{\beta }\vec{\beta }+w_{i}\vec{\lambda }\right]$ are passed to BART. Then, the BART sampler produces a draw of each tree, $M_{j},T_{j}|\vec{Y},{\mathrm{vec}}_{i}\left[x_{i}^{\beta }\vec{\beta }+w_{i}\vec{\lambda }\right],\sigma ,M_{-j},T_{-j}$. ${\mathrm{vec}}_{i}f\left(x_{i},z_{i};\vec{M},\vec{T}\right)$ is passed back to Stan, completing the cycle. This proceeds from starting points sampled from the prior distribution over BART trees with the offset and variance estimated from a linear or binary, multilevel model maximum likelihood fit, repeats through a warm-up phase during which the Stan sampler performs adaptation of its proposal distribution, and finally iterates through the set of samples from the posterior that are intended for inference. While this strategy is similar to a similar proposal [50], our approach allows the same covariates in the parametric and nonparametric components and has a shareable software implementation.

### 4.5. stan4bart Model Specification

Individual level covariates can enter a stan4bart model in the parametric mean component, the nonparametric mean component, or both. Parametric terms for covariates that are not included in the nonparametric component of the model have the benefit of being interpretable as Bayesian multilevel regression coefficients with the downside of potentially requiring nonlinearities and interactions to be explicitly specified. On the other hand, exclusively nonparametric terms are more flexible, but suffer from reduced explicability [51].

The pros and cons of including covariates in both components of the model are not clear-cut, however, we can consider some use cases. For instance, suppose we know that some of the covariates are particularly important for predicting the outcome, but we are unsure that the relationship will be easily captured by a parametric model. We may also believe that the stronger a continuous covariate’s association with the outcome, the harder it is to accurately approximate its true relationship to the outcome with step functions and regression trees. In that case, including such a covariate in both model components may have computational benefits because it may simplify the nonparametric model, which now just has to account for the part of the response surface that *is not* linear. This specification might lead to faster convergence and, potentially, more precise estimates.

On the other hand, what if we are fairly confident in our specification of a parametric model for some covariates? In that case, one might wonder what could be gained from additionally including one or more of the covariates from that parametric model in the nonparametric component. However, in this setting, including a covariate in both components is an example of *parameter expansion*, a technique often employed in Gibbs samplers to reduce dependence between parameters and increase the efficiency of the sampler [52,53,54]. In such a case, neither the parametric nor the nonparametric components would be directly identifiable but crucially their sum would still be. Thus, while we might not strive to overparametrize, we are hopeful it need not be problematic if we do. More research will need to be performed to confirm this.

Consequently, we offer the following practical guidance on how to include predictors in stan4bart models:

- If a parameter *must* be interpreted as a regression coefficient or if the functional form of its relationship to the response is known, include it only in the parametric component.
- Otherwise, include all individual predictors in the nonparametric component.
- Consider including strong predictors or ones that are substantively associated with the outcome in both components, but be mindful that in doing so, the *linear model coefficients are not directly interpretable*.
- Users who are comfortable with the above caveat can center their model on a simple linear regression, so that BART effectively handles only the non-linearities in the residuals of that fit.

### 4.6. stan4bart Software

The stan4bart package in R, available on the Comprehensive R Archive Network (CRAN), provides a user-friendly, multithreaded implementation of the algorithm above. Models are specified by using the following language constructs, chosen to be familiar to users of other R software packages:

- The R standard left-hand-side–tilde–right-hand-side formula construct gives the base of a parametric linear model, for example, response ∼ covariate_a + covariate_b + covariate_a:covariate_b.
- Multilevel structure is included by adding to the formula, terms of the form (1 + covariate_c | grouping_factor), where the left-hand side of the vertical bar gives intercepts and slopes, while the right-hand side specifies the variable across which those values should vary. The full set of syntax implemented is described in Bates et al. [44].
- The BART component is specified by adding to the formula, a term of the form bart(covariate_d + covariate_e). In this case, the “+” symbol is symbolic, indicating the inclusion of additional variables among those eligible for tree splits.

As a convenience, a “.” can be used to specify all available variables, and subtraction (“-”) can be used to remove variables from that set. A typical shorthand for fitting a causal model with varying intercepts and slope for treatment would be specified similar to the formula response ∼ treatment + bart(. - group) + (1 + treatment | group).

## 5. BART and stan4bart for Causal Inference

It is straightforward to use BART and stan4bart to estimate any of a variety of average treatment effects under the assumptions above. We first describe the standard BART implementation and then discuss the additional modeling choices that arise when using stan4bart.

### 5.1. BART for Causal Inference

When using BART for causal inference the first step is to fit BART to the observed data, that is, the outcome given the treatment indicator and covariates. Based on evidence from simulations and previous data analysis challenges, we recommend running 8 to 10 chains for each BART fit [55] and checking convergence using a statistic that is meaningful for the desired estimand (such as the SATT estimate [37]).

The model fit can be used to make predictions for two counterfactual datasets [1]. The covariates are kept intact for both; however, in one, all treatment values are set to 0, and in the other they are all set to 1. This allows BART or stan4bart to draw from the posterior distribution for E[Y(0)∣X=x] and E[Y(1)∣X=x] for each person, meaning that we can also obtain draws from E[Y(1)−Y(0)∣X=x], the iCATE for each person. Various combinations of these posterior distributions and the observed data can then be used to obtain posterior distributions of average treatment effects either for the full dataset or any subset thereof, and for sample, condition, and population quantities.

For example, consider the SATT estimand. Our best guess of $Y_{i}\left(1\right)$ for anyone in the treatment group is simply their observed outcome, $Y_{i}$. Our estimate of $Y_{j}\left(0\right)$, however, is the mean (or median) draw from the posterior predictive distribution for the counterfactual outcome for individual *i* in group *j*, ${\tilde{Y}}_{i}\left(0\right)$. We can thus define a new quantity ${\tilde{\tau }}_{i}$ to be the draw of the individual treatment effect for individual *i* from its posterior predictive distribution. Averages of these draws can be used to estimate the SATT. More specific subsets of this summation can be used to estimate any subgroup estimand of interest including the SGATE, SGATT, and iCATEs defined above.

The R package bartCause (available on CRAN) provides a handy wrapper function for the dbarts implementation of BART and stan4bart that simplifies the process of using BART for causal inference by implementing the fitting and prediction steps described above and by setting the defaults for the prior specification and model fitting (number of chains, iterations per chain, etc.) to values found to be useful in practice. It is straightforward to make inferences about any of the estimands described in this article either as estimates and confidence intervals or draws from the (Monte Carlo approximation to the) relevant posterior or posterior predictive distribution.

### 5.2. stan4bart for Causal Inference

To use stan4bart for causal inference, we can also use the algorithm directly. The key is to specify the model so that it is possible to extract information about the appropriate estimands. There are now two additional parametric pieces of the model to specify, however, $x^{\beta }\vec{\beta }$ and $w\vec{\lambda }$. As described above, we advise parsimony when specifying $x^{\beta }\vec{\beta }$. It should be used for predictors that have special significance (for instance, the treatment variable in a causal analysis), predictors (or transformations thereof) suspected to have a linear relationship with the outcome, or suspected moderators. $w\vec{\lambda }$ captures intercepts and slopes that vary across groups.

Suppose you wanted to fit a model for causal inference, assuming that the response variable, y, treatment variable, z, and a grouping variable, g, are in a data frame data together with any additional confounders The following code demonstrates how to specify the stan4bart function to estimate treatment effects in a setting where you suspect that observations are correlated within groups (operationalized as g).

# varying intercepts

# we will train the model on the observed data in "data"

# but we also need to construct a dataset, "data.test",

# we use data.test to obtain counterfactual predictions

data.test <- data

data.test$z <- 1 - data.test$z

fit <- stan4bart(

# this next line only includes varying intercepts

y ~ z + bart(. - g) + (1 | g),

train = data,

test = data.test

)

 

To fit a stan4bart model that additionally accommodates varying slopes, the group structure term can be altered as follows to account for varying slopes across groups:

 

# varying intercepts and slopes

# this code is similar to above in creating training and

# test datasets

data.test <- data

data.test$z <- 1 - data.test$z

fit <- stan4bart(

# this next line includes the varying slopes for z

y ~ z + bart(. - g) + (1 + z | g),

train = data,

test = data.test

)

 

stan4bart has been integrated into the bartCause package for ease of use producing estimates of a variety of causal estimands. However, they can be manually extracted in the following manner:

 

## CATE

# Each draw is from the posterior of the expected value

# of the response under the observed and counterfactual

# treatment conditions.

 

# Matrices of size: n.observations x n.samples

mu.obs.samples <- extract(fit, sample = "train")

mu.cf.samples  <- extract(fit, sample = "test")

 

z <- data$z

mu.1.samples <- z * mu.obs.samples + (1 - z) * mu.cf.samples

mu.0.samples <- (1 - z) * mu.obs.samples + z * mu.cf.samples

 

icate.samples <- mu.1.samples - mu.0.samples

cate.samples <- rowMeans(icate.samples)

 

# Estimands

cate <- mean(cate.samples)

cate.lb <- cate - 1.96 * sd(cate.samples)

cate.ub <- cate + 1.96 * sd(cate.samples)

 

## SATE

# Draw from the posterior predictive distribution.

y.obs <- data$y

y.cf.samples <- extract(fit, sample = "test", value = "ppd")

 

y.1.samples <- z * y + (1 - z) * y.cf.samples

y.0.samples <- (1 - z) * y + z * y.cf.samples

 

ite.samples <- y.1.samples - y.0.samples

sate.samples <- rowMeans(ite.samples)

 

sate <- mean(sate.samples)

sate.lb <- sate - 1.96 * sd(sate.samples)

sate.ub <- sate + 1.96 * sd(sate.samples)

 

To obtain intervals and estimates for effects on the treated population, subset the individual effect matrices prior to averaging across rows.

### 5.3. Fixed vs. Random Effects

It is worth noting that we assume that our causal assumptions have not changed from above. That is, the grouping variables are not acting as confounders, they impact only the error structure of the data generating process (henceforth, DGP). Of course, in practice, in any given setting, it is always possible that ignorability would not be satisfied solely given the other covariates but would be satisfied when conditioning on the grouping variable as well. In that case it might be helpful to include the grouping variable as a fixed effect as well, since the random effects assumption would not be expected to hold, and conditioning on group level fixed effects allows one to control for any unmeasured group level confounders. In the most likely scenario that ignorability is not satisfied even conditional on the grouping variable—that is, there are unmeasured individual level confounders—a random effects specification tends to be a reasonable compromise between ignoring the group level structure entirely and using fixed effects, as fixed effects can act as bias-amplifying covariates [56,57].

## 6. Simulation Design

We designed a set of simulations to better understand the properties of stan4bart relative to close alternatives that either (1) have parametric assumptions or (2) cannot explicitly accommodate more general error structures. This section outlines our simulation design which has the general goal of trying to mimic a realistic data structure.

### 6.1. Original IHDP Simulation

The basic structure of our simulation mimics the simulation structure developed by Hill [1] in the paper that first introduced machine learning for causal inference. This simulation used data from a randomized experiment called the Infant Health and Development Program (IHDP; [58,59]) conducted in the 1980s to understand whether intensive childcare in the first few years of life could have a positive impact on the development of children who were born low-birth-weight and premature.

This study randomized roughly one third of the 985 participating families to participate in the IHDP intervention. Participants were eligible for intensive, high-quality child care and home visits from a trained provider during the first three years of infancy. A subset of the covariates collected during the baseline phase of that study and used frequently in subsequent evaluations of the IHDP program were included as the covariates for that simulation. Thus, the simulation reflected the actual distributions for and associations among covariates found naturally in existing data. The simulation covariates comprised six continuous, nine binary, and two unordered categorical variables reflecting child measurements at birth, the mother’s sociodemographic characteristics at the time of birth, behaviors engaged in during pregnancy, and indicators for the study site.

To construct an observational study for the simulation, a hypothetical treatment assignment was induced by removing a nonrandom portion of the originally randomized treatment group, those children born to nonwhite mothers. This destroyed the independence between the originally randomized treatment assignment and the covariates but maintained the common support for the new treatment group. By simulating outcomes for the remaining sample with a mean structure that was a function solely of the treatment and covariates, ignorability was satisfied by construction.

To explore the ability of BART to flexibility fit nonlinear response surfaces, three different DGPs were used to generate potential outcomes. Response surface A was linear for both E[Y(0)∣X=x] and E[Y(1)∣X=x] and had a constant treatment effect. Response surface B created heterogeneous treatment effects by keeping the model for E[Y(0)∣X=x] linear but allowing the model for E[Y(1)∣X=x] to be nonlinear by exponentiating a linear combination of the covariates. Response surface C created heterogeneous treatment effects by including a variety of squared terms and interactions.

In the original paper [1], this simulation was used to demonstrate the superior performance of BART for causal inference relative to linear regression and a generic implementation of propensity score methods. Since Hill [1] was published, testing grounds have been developed that allow for comparisons between BART and propensity score methods, in which the propensity score methods were able to be more carefully curated by methodologists who were experts in that field. These have also shown superior performance of BART [15]. While that paper also explored performance in settings where the common support assumption was violated, the current study restricted attention to scenarios where common support was satisfied to allow space for exploring features of the data specific to multilevel settings.

### 6.2. Extensions to the Original IHDP Simulation

This section details how we extended the original IHDP simulation to allow for a group structure and explore other features of the DGP.

#### 6.2.1. Adding Group Structure to the Response Surfaces

We wanted to create a grouped structure that would mimic those features of a grouped data structure that exist naturally. Therefore, we repurposed two variables that were used as covariates in the original IHDP simulation and treated them as grouping variables in the current simulation. The first of these was the collection of eight indicators for the study site (a blocking variable in the original IHDP experiment). The other was a variable representing the mothers’ age at birth (treated as continuous in the original simulation) which had 26 levels.

These groups were incorporated into the response surface in two different ways. The **varying intercepts** setting generated data from the respective response surface

$$\begin{array}{ccc} Y_{i}\left(0\right)|\lambda_{g[i]}^{\mathrm{int}},\epsilon_{i}^{0} & = & h^{z}\left(x_{i}\right)+\lambda_{g[i]}^{\mathrm{int}}+\epsilon_{i}^{0},\\ Y_{i}\left(1\right)|\lambda_{g[i]}^{\mathrm{int}},\epsilon_{i}^{1} & = & h^{z}\left(x_{i}\right)+\lambda_{g[i]}^{\mathrm{int}}+\tau^{*}+\epsilon_{i}^{1},\\ \lambda_{g}^{\mathrm{int}} & ∼ & N(0,\sigma_{\lambda^{\mathrm{int}}}),\\ \epsilon_{i}^{0} & ∼ & N(0,\sigma_{0}),\\ \epsilon_{i}^{1} & ∼ & N(0,\sigma_{1}), \end{array}$$

where $h^{z}\left(x_{i}\right)$ reflects the function of the covariates specific to the given potential outcome and either response surface A, B, or C. $\tau^{*}$ only appears in the model for Y(1) and represents the constant treatment effect when $h^{0}\left(x_{i}\right)=h^{1}\left(x_{i}\right)$ in response surface A. In response surface B and C, these are not equal an thus heterogenous treatment effects that vary with levels of the covariates are induced. The asterisk is meant to remind the reader that $\tau^{*}$ should not necessarily be interpreted as a constant or average treatment effect. $\lambda_{g}^{\mathrm{int}}$ is the varying intercept that corresponds to the grouping variable in question.

In contrast, the **varying intercepts and slopes** setting generated data from an augmented version of the above

$$\begin{array}{ccc} Y_{i}\left(0\right)|\lambda_{g[i]}^{\mathrm{int}},\epsilon_{i}^{0} & = & h^{z}\left(x_{i}\right)+\lambda_{g[i]}^{\mathrm{int}}+\epsilon_{i}^{0},\\ Y_{i}\left(1\right)|\lambda_{g[i]}^{\mathrm{int}},\lambda_{g[i]}^{\mathrm{slo}},\epsilon_{i}^{1} & = & h^{z}\left(x_{i}\right)+\lambda_{g[i]}^{\mathrm{int}}+\lambda_{g[i]}^{\mathrm{slo}}+\epsilon_{i}^{1},\\ \lambda_{g}^{\mathrm{int}} & ∼ & N(0,\sigma_{\lambda^{\mathrm{int}}}),\\ \lambda_{g}^{\mathrm{slo}} & ∼ & N(0,\sigma_{\lambda^{\mathrm{slo}}}),\\ \epsilon_{i}^{0} & ∼ & N(0,\sigma_{0}),\\ \epsilon_{i}^{1} & ∼ & N(0,\sigma_{1}). \end{array}$$

This specification allowed the model for Y(1) to include the term $\lambda_{g[i]}^{\mathrm{slo}}$ rather than the $\tau^{*}$ in the varying intercepts specification so that treatment effects could vary explicitly by group according to a distribution of varying slopes.

The choice of grouping variable and whether or not the varying slopes were included in the DGP represented two distinct simulation knobs, each with two levels. Combined with the three response surfaces discussed above, this created 12 different settings within which to evaluate performance.

#### 6.2.2. Additional Simulation Knobs Explored

We also explored the variation in performance across two settings that are not represented in the results in the next section for the sake of parsimonious exposition. First, we assessed the variation in performance based on the size of the treatment effect. Expressed in units standardized by the standard deviation of the outcome, these effect sizes we examined were 0, 0.2, 0.5, and 0.8. We found no difference in results across these choices. We also tested the differences in performance based on intraclass correlation values of 0.2, 0.333 and 0.5. We also found no difference in results across these choices.

### 6.3. Methods Compared

We compared the performance of a variety of methods in an attempt to understand the advantages of combining flexible modeling with the ability to explicitly incorporate more complicated grouped error structures.

#### 6.3.1. Linear Models

We fit several linear models to the data. **Linear full pool** is a linear regression where the groups are ignored entirely. **Linear f.e.** is a linear regression with fixed effects included for the grouping variables; this represents our no pooling option. **Linear v.i.** is a linear regression with varying intercepts. **Linear v.i.s** is a linear regression with varying intercepts and varying slopes, where the slopes in question are the coefficients on the treatment variable. Each of the last two were fit using the stan_lmer function in rstanarm.

Given that the group-level estimands were one of our areas of focus it seemed unfair to not include versions of the above that more explicitly targeted these estimands. We included two additional models with this in mind. **LinearX f.e.** is a standard linear regression that includes both fixed effects and interactions between the fixed effects and the treatment variable. **LinearX v.i.s.** is an implementation of the stan_lmer function that allows for both varying intercepts and varying treatment effects.

#### 6.3.2. BART-Based Models

We also fit several different versions of BART models. **vanilla BART** uses a traditional BART specification similar to that used in Hill [1] but specifically omitting the grouping variables and including the propensity score as a covariate. **BART f.e.** extends this basic implementation by adding fixed effects for the grouping variables. **BART v.i.** is a BART implementation that allows for varying intercepts through the rbart_vi function in dbarts. All BART implementations included a propensity score as suggested by Hahn et al. [7]. The propensity score was estimated using BART using a hyperprior on the end-node variance, making it extremely unlikely to take on small values and thus overfit, essentially guarding against the problems induced by the originally proposed implementation [37]. Finally, we also implemented Bayesian causal forests, which we denote **vanilla BCF** and **BCF f.e.**

#### 6.3.3. stan4bart Implementations

We implemented two different versions of stan4bart. The simpler version, **stan4bart v.i.**, allows for varying intercepts. The slightly more complicated version, **stan4bart v.i.s.**, allows for varying intercepts and slopes.

To fit **stan4bart v.i.**, models with varying intercepts were specified as:

 

fit <- stan4bart(

y ~ bart(. - g) + (1 | g),

train = data,

test = data.test

)

 

Fitting **stan4bart v.i.s.** allowed for a variation in both intercepts and slopes and was specified as:

 

fit <- stan4bart(

y ~  bart(. - g) + (1 + z | g),

train = data,

test = data.test

)

## 7. Simulation Results

We compared methods based on performance with respect to several criteria for each of our targeted estimands. We present the results for each estimand in turn.

### 7.1. SATT

We evaluated the performance with respect to SATT for each of our methods across the six different settings by focusing on the root-mean-square error (RMSE), average interval length, and coverage. The RMSE and interval length were standardized by the standard deviation of the outcome variable so that the absolute size of each measure was more meaningful.

**Figure 1:**
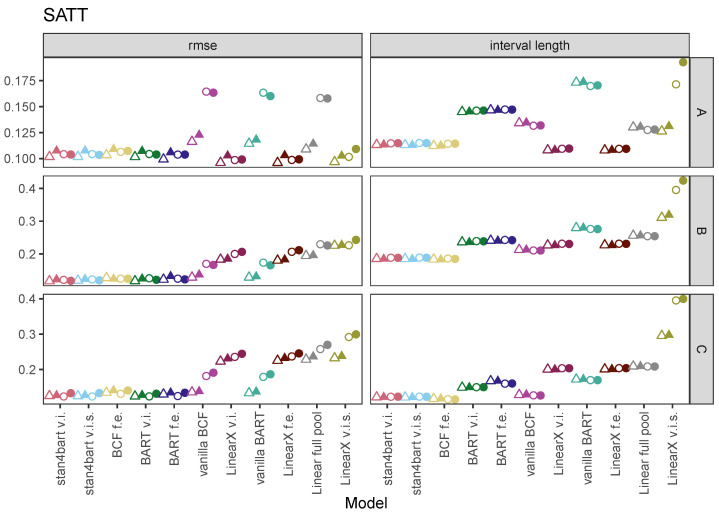
Results of our simulations for each method with respect to SATT as measured by RMSE (**left** panel) and average interval length (**right** panel). Each row corresponds to one of the three response surfaces (A, B, or C). Shapes are used to represent one of two grouping structures, triangles are for results from grouping structure 1, and circles for results from grouping structure 2. Hollow shapes represent results from DGPs with random intercepts and solid shapes represent results from DGPs with random intercepts and random slopes.

Figure 1 displays the results of our simulations for each method with respect to SATT as measured by RMSE (y-axis) and the average interval length using six plots. Rows correspond to response surfaces (A, B, or C) and columns to the metric displayed (RMSE or interval length). The results specific to the choice of grouping variable (group 1 or group 2) are displayed on each plot with different shapes (triangle or circle, respectively). The grouping structure is represented by whether the plotted shape is hollow (varying intercept) or filled (varying intercept and slope).

The results for the linear response surface (A) demonstrate strong performance overall from all methods with regard to RMSE with the possible exceptions of the vanilla BART and BCF implementations and the pooled linear regressions in the group 2 version of the DGPs. Given the simplicity of the response surface, these results are not surprising—the only complexity is the grouping structure. The differences across methods are more apparent in the average interval lengths. Here, the linear models that allow for variation (either intercept or slope) have the shortest intervals followed by the BCF and stan4bart methods. These are followed by BCF with fixed effects and linear regression, and then the BART methods with fixed effects and varying effects. The BART implementations that completely ignore the grouped variables not surprisingly performs the worst overall on this metric. One odd result is the linear model with varying slopes, which performs reasonably well with regard to the interval length for the first grouping variable but much worse for the second. We suspect that this has to do with the fact that while the group 1 version has more levels, the correlation structure of group 2 is more complex. The effect of different correlation on the performance of different methods is beyond the scope of this paper but is an issue that could be explored in future simulation studies.

The ordering with regard to performance changes for some methods once we move to the results for the nonlinear response surfaces in the second and third rows. These are more challenging for all of the methods (note the change in the y-axis) but particularly for those that have strict linear parametric requirements. The strongest consistent performers with regard to RMSE are the stan4bart methods, **BCF f.e.**, **BART v.i.**, and **BART f.e.**. The versions of BCF and BART that ignore the group structure perform fine in the setting with the first grouping variable (triangles) but less well with the second (circles). The linear models perform the worst. The best performers with regard to the average interval length are again the flexible fitters with an edge once again for the stan4bart and BCF methods.

The performance with regard to the interval length for response surfaces B and C highlights the differences between the stan4bart methods and **BCF f.e.** relative to vanilla BCF (with just slightly longer intervals) and the BART methods with grouping structure. The linear methods trail with **LinearX v.i.s.**, demonstrating by far the longest intervals. Vanilla BART has longer intervals than the best linear models for response surface B and slightly longer ones for response surface C.

**Figure 2:**
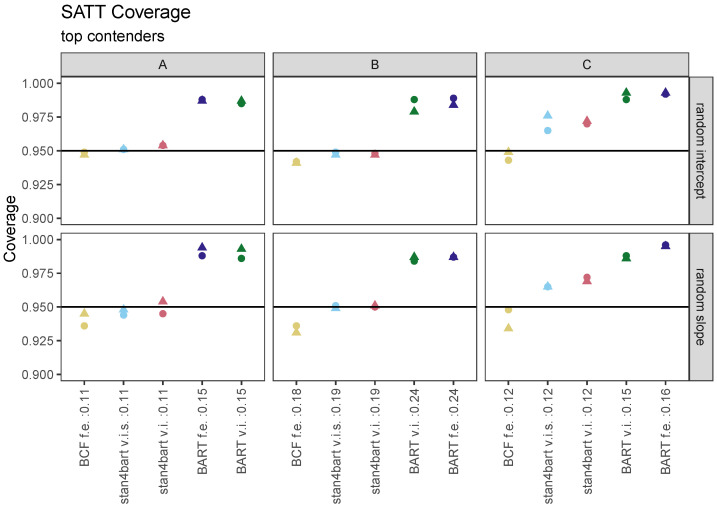
Percentage of 95% intervals that covered the true SATT for each of the top-performing methods. Results are presented separately by settings defined by response surface (columns A, B, C) and multilevel structure (rows: varying intercepts or varying intercepts and slopes). Results from settings defined by grouping variable are displayed on the same plot with different symbols. Labels on the x-axis additionally provide the average interval length (across both grouping settings).

A shorter interval length is only an asset, however, if nominal coverage is achieved. Figure 2 displays the coverage results for the top contenders across our 12 settings. In addition, the plots include the average interval length across grouping settings for each response surface as part of the method label on the x-axis. These plots indicate that the stan4bart methods seem to strike the best balance between having a low RMSE and shorter intervals while still maintaining nominal coverage. The BCF methods which performed similarly to the stan4bart methods with regard to the RMSE and interval length struggled a bit more to achieve nominal coverage, particularly for response surface B.

### 7.2. GSATT

The results for the group-level ATTs are more complicated because we have many more estimands to consider (one for each group). Thus, we organized the plots to display the RMSE and interval length results on separate plots. Since there was virtually no distinction in the results between the two grouping settings—varying intercept versus varying intercept and slope—we elected to collapse those results. Instead, we broke out our group 1 and group 2 results into separate sets of plots (top and bottom panels).

**Figure 3:**
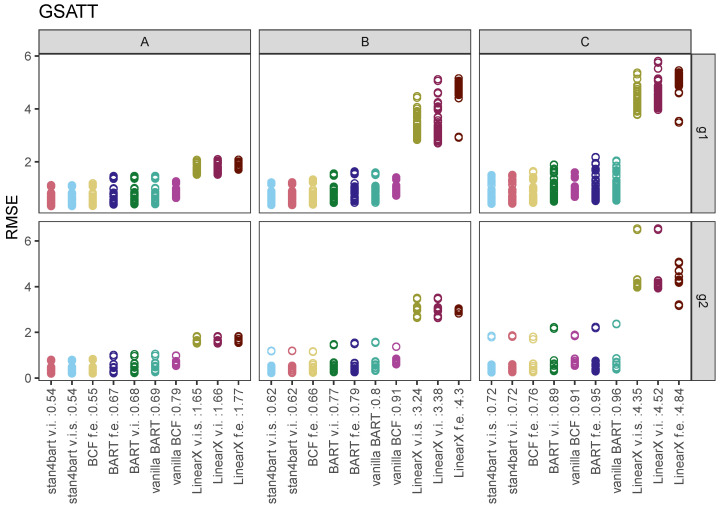
RMSE results for the group-level estimands across methods. Each plot corresponds to a setting defined by grouping variable (row) and response surface (column). Results are collapsed across the settings defined by varying intercepts versus varying intercepts and slopes. Average RMSE across these settings and across estimands are displayed numerically next to the name of each method, separately for each response surface. Estimands that were covered by a 95% interval produced by the method were filled in rather than left hollow.

Figure 3 displays the RMSE for each method (x-axis) and group-level estimand across the six settings defined by the response surface (columns) and grouping variable (rows). The performance for each method is displayed in its own column with separate points for each estimand (group-level ATT). The average RMSE across estimands for each method is displayed next to the label for its name for each response surface (collapsed across settings defined by grouping variable). Across all of the response surfaces, the stan4bart methods perform the best followed very closely by **BCF f.e.** and then the other BART-based methods. The linear methods perform noticeably worse in all settings but in particular when the response surface is nonlinear (B) and additionally when the treatment effects are heterogeneous by covariate values (C).

**Figure 4:**
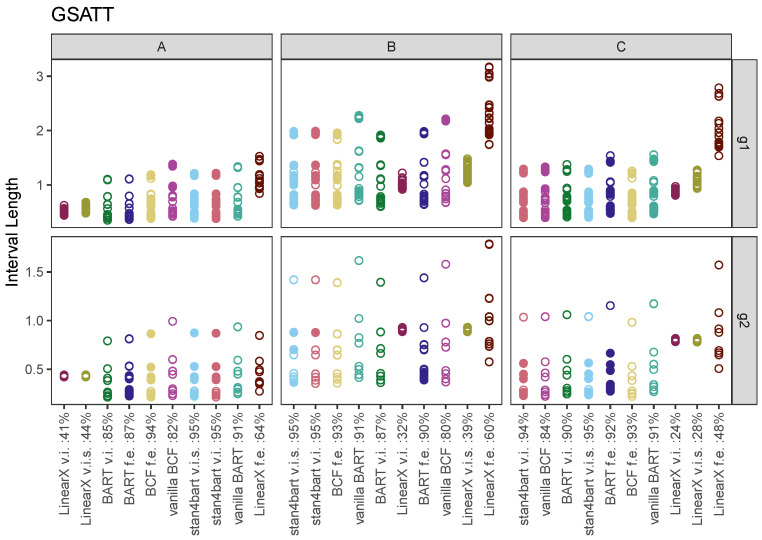
Interval length results for the group-level estimands across methods. Each plot corresponds to a setting defined by grouping variable (row) and response surface (column). Results are collapsed across the settings defined by varying intercepts versus varying intercepts and slopes. Average coverage across these settings and across estimands are displayed numerically next to the name of each method, separately for each response surface. Within each vertical panel the methods are ordered by average interval length across both grouping variable settings and estimands. Estimands that were covered by a 95% interval produced by the method were filled in rather than left hollow.

Figure 4 displays the average interval length for each method (*x*-axis) across the six settings defined by the response surface (columns) and grouping variables (rows). The performance for each method is displayed in its own column with separate points for each estimand (group-level ATT). The results that achieved nominal coverage for a given estimand are displayed with solid rather than open circles for each group-level estimand.

The average coverage for each method and response surface combination (collapsed across other sources of variability) is displayed next to the name of each method. For response surface A, the linear methods with varying intercepts and slopes have the shortest intervals; however, the coverage with respect to the group estimands is quite poor, averaging 41% and 44%. The interval length for these methods increases with the more complicated response surfaces and in the scenarios with the first group variable is more variable across group estimands. **LinearX f.e.** performs the worst in terms of interval length but has better coverage properties across the board.

The other methods perform reasonably similarly with regard to the distribution of interval lengths across group-level estimands; however, the stan4bart implementations and **BCF f.e.** are also able to maintain the best coverage. **stan4bart v.i.s** is the only method that achieves nominal average coverage across all three response surfaces and **vanilla BCF** performs the worst in this regard with an average coverage dipping to 80% for response surface B.

**Figure 5:**
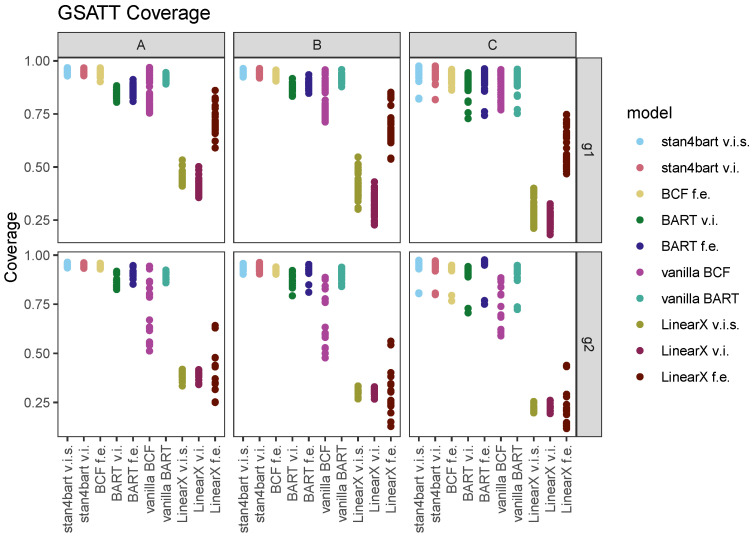
Coverage rates for each method with respect to each of the group-level estimands. Plots vary by settings defined by grouping variable (rows) and response surface (columns A, B, and C) and are collapsed across grouping scenarios (varying intercept versus varying intercept and slope).

Figure 5 displays the coverage percentages separately for each combination of method, grouping variable, and estimand and is thus capable of revealing greater distinctions across methods that looked similar in the previous plot. With one exception, the stan4bart demonstrate the least variability in coverage rates across groups. **vanilla BCF** has the greatest variability in coverage among the flexible models. **LinearX t.e.** is unable to provide reasonable coverage in the setting with the second grouping variable; however, it performs far better with respect to the covariate of group-level estimands than the other linear methods for the setting defined by the first grouping variable.

### 7.3. iCATEs

We evaluated the ability of each method to estimate the CATE for each combination of covariate values that manifested in each sample as the iCATEs. To compare performance, we used the metric proposed in Hill [1], the precision in estimation of heterogeneous effects measure, or PEHE. This was calculated within each dataset for a given method as the square root of the average of the squared differences between the estimate of the iCATE and the true iCATE for each person.

**Figure 6:**
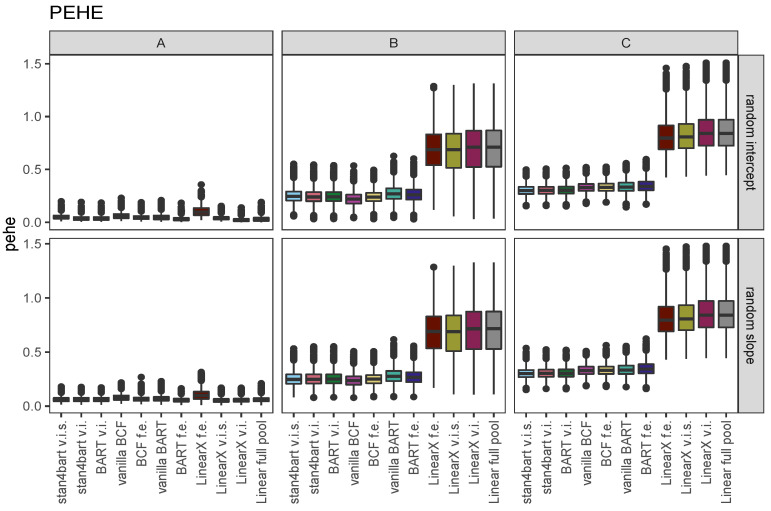
PEHE results for each of the methods across the six settings defined by response surface (columns A, B, and C) and multilevel setting (rows corresponding to varying intercepts versus varying intercepts and slopes). Results are collapsed across the DGPs defined by the grouping variable.

Figure 6 displays the PEHE results for each of the methods across the six settings defined by response surface and multilevel setting (varying intercepts versus varying intercepts and slopes). Results are collapsed across the DGPs defined by the grouping variable.

For the linear response surface A, which has a constant treatment effect, all of the methods perform similarly which is not surprising given the ease of the task. The only method that noticeably performs a bit worse is the linear model with fixed effects interacted with the treatment, likely because it is overfitting. The landscape changes for the nonlinear response surfaces where the top performing methods are the flexible models with the strongest performance demonstrated by the stan4bart methods, BCF with fixed effects, and BART with varying intercepts.

## 8. Discussion

The goal of this work was to develop a method that could extend the BART framework for the flexible fitting of response surfaces to accommodate more complex error structures. We evaluated the utility of this approach by assessing performance in a causal inference context that allowed for varying intercepts or varying intercepts and slopes. For one of our three response surfaces, this heterogeneity was in addition to the heterogeneity in treatment effects that was a systematic (nonrandom) function of observed confounders.

Our results indicated that the stan4bart models provided superior performance when compared against both methods with flexible fit that did not allow for a more complicated error structure as well as methods that explicitly accommodated a grouped error structure but assumed a linear parametric mean structure. Throughout, BCF was a strong competitor on all performance measures even though it did not explicitly accommodate the error structure.

We evaluated stan4bart in a causal setting, which is generally more challenging than standard prediction settings. Given its strong performance in this challenging setting, we recommend the use of stan4bart both in causal and noncausal settings. More broadly, we hope that stan4bart will be a jumping-off point for the further development of methods that aim to marry flexible mean structures with parametric approaches to either the mean structure or the grouped error structure.

## Data Availability

This data can be found here: https://github.com/gperrett/stan4bart-study (accessed on 14 August 2022).
